# Hybrid Breast Reconstruction Revisited: Patient-Reported Outcomes Following Fat Grafting

**DOI:** 10.3390/jcm15031158

**Published:** 2026-02-02

**Authors:** Ioan Constantin Pop, Maximilian Vlad Muntean, Vlad Alexandru Gata, Radu Alexandru Ilies, Delia Nicoara, Claudiu Ioan Filip, Vasile Pop, Patriciu Andrei Achimas-Cadariu

**Affiliations:** 1 Faculty of Medicine, “Iuliu Hațieganu” University of Medicine and Pharmacy, 400012 Cluj-Napoca, Romaniailies.radu.alexandru@elearn.umfcluj.ro (R.A.I.);; 2 Department of Plastic Surgery, “Prof. Dr. I. Chiricuță” Institute of Oncology, 400015 Cluj-Napoca, Romania; 3 Department of Plastic and Reconstructive Surgery, “Iuliu Hațieganu” University of Medicine and Pharmacy, 400012 Cluj-Napoca, Romania; 4 Department of Surgical Oncology and Gynecologic Oncology, “Iuliu Hațieganu” University of Medicine and Pharmacy, 400012 Cluj-Napoca, Romania; 5 Department of Surgical Oncology, “Prof. Dr. I. Chiricuță” Institute of Oncology, 400015 Cluj-Napoca, Romania; 6 Department of Quality Management, “Prof. Dr. I. Chiricuță” Institute of Oncology, 400015 Cluj-Napoca, Romania; 7 First Surgical Clinic, Faculty of Medicine, “Iuliu Hatieganu” University of Medicine and Pharmacy, 400012 Cluj-Napoca, Romania; 8 Department of Plastic Surgery and Burn Unit, Emergency District Hospital, 400535 Cluj-Napoca, Romania; 9 Department of Oral and Maxillofacial Surgery, Bihor County Emergency Hospital, 410169 Oradea, Romania

**Keywords:** aesthetic outcome, autologous fat grafting, breast implant, hybrid breast reconstruction, implant-based reconstruction, lipofilling

## Abstract

**Background/Objectives**: Hybrid breast reconstruction (HBR), which combines implant-based reconstruction with autologous fat grafting (lipofilling), has emerged as a promising approach for improving both aesthetic and functional outcomes following mastectomy. This study aimed to evaluate patient-reported outcomes using the BREAST-Q questionnaire before and after lipofilling, focusing on aesthetic satisfaction, physical well-being, and quality of care. **Methods**: This before–after study included patients who underwent prepectoral implant-based reconstruction followed by one session of lipofilling between November 2024 and May 2025. The BREAST-Q questionnaire was administered preoperatively and at three months postoperatively. Statistical analyses were conducted to compare changes across aesthetic, functional, and care-related domains. **Results**: A total of 96 patients completed both pre- and postoperative questionnaires. Statistically significant improvements (*p* < 0.01) were observed in most aesthetic and psychosocial parameters, including satisfaction with breast appearance (Q1), psychosocial well-being (Q2), sexual well-being (Q4), and satisfaction with surgical outcomes (Q5). Physical symptoms such as discomfort (Q3) decreased significantly postoperatively. Satisfaction with medical care remained high, with minor improvements noted. No oncologic recurrences were reported. **Conclusions**: Hybrid breast reconstruction using fat grafting after implant placement offers significant benefits in terms of aesthetics, symptom relief, and patient satisfaction. It is a safe and effective procedure that can be widely integrated into clinical practice, provided that patient selection and technique are carefully considered.

## 1. Introduction

Recently, the expectations of patients undergoing breast surgery have increased, where naturality is continuously gaining interest. Total mastectomy, due to its aggressivity and clear postoperative results, can be considered as devastating for many patients who are affected by breast cancer. Breast reconstruction is of the utmost importance in terms of emotional and physical recovery for this category of patients. It can be realized in two different ways—using prosthetic implants (implant breast reconstruction) or respectively autologous tissue harvested from the patients (known as autologous breast reconstruction). For the latter, DIEP, TRAM and latissimus dorsi flaps remain the major techniques which are applied, with encouraging results for some categories of patients. Whatever the surgical technique is, it can be realized immediately after mastectomy or delayed, as patients sometimes undergo adjuvant chemotherapy and radiotherapy [1,2].

Implant-based breast reconstruction is one of the most popular methods, which is a type of prosthetic reconstruction. The current tendency is to switch from the traditional retropectoral (submuscular) technique with the prepectoral approach. Hybrid breast reconstruction combines several techniques such as skin expansion, multiple sessions of fat grafting and the use of implants [3].

Lipofilling, also known as autologous fat grafting, is a procedure which makes use of the patient’s own adipose tissue to enhance the volume, contour and symmetry of various anatomic regions, with high utilities in the field of breast surgery. Despite being a widely applied technique nowadays, its advantages may be underestimated—it can radically change the appearance of the body contour, whatever the injected region is. It consists of harvesting fat from various regions (like the abdomen, thighs, and buttocks) [4,5]. Furthermore, fat is carefully purified, ensuring optimal quality for transfer [6]. After this, fat is injected into the receptor area, commonly the breasts and the face. It provides the breasts with a natural appearance and consistency, optimizing their overall aspect [1].

When applied as an adjuvant procedure for breast reconstruction with implant, lipofilling can be a key factor in terms of aesthetic results, because it has systematically been shown that hybrid breast reconstruction has better results than breast reconstruction with implant alone [7].

Beyond its aesthetic indications which are clear, lipofilling has some medical advantages, too. The risk of allergic reactions and even rejection is reduced, due to its autologous character. It has been reported that fat grafting is able to reduce post-radiotherapy capsular contracture, helping the process of wound healing and, therefore, antagonizes the fibrotic effect that radiotherapy has in the area where it is applied [3,7]. Lipofilling can also be applied in the treatment of postmastectomy pain syndrome, another complication which might be met in the field of breast surgery. It also reduces pain caused by capsular contracture [1,8]. Moreover, the vascular plexus which is developed in the receptor region can improve overall skin quality [4,5].

Lipofilling is known for providing benefits with a low rate of complications, being considered a safe procedure nowadays [9,10]. However, its safety had been questioned from an oncological point of view. It had been thought that it may help the spread process of malignant cells, gaining oncogenic potential. Various studies disprove this, stating that it is considered safe to apply, and this theory was invalidated [11,12].

This is a prospective cohort study designed to evaluate the clinical benefits and safety profile of autologous fat grafting (AFG) when used as an adjunct to implant-based breast reconstruction (hybrid reconstruction). The objective of this study is to assess the impact of AFG on clinical outcomes, aesthetic results, and patient satisfaction in breast reconstruction.

## 2. Materials and Methods

The type of this study is a before–after study (pre–post analysis) evaluating patient-reported outcomes following fat grafting in hybrid breast reconstruction. Patients who underwent prepectoral implant-based breast reconstruction and subsequently received a secondary lipofilling session as part of the staged reconstruction process were found eligible for this study. Pure autologous reconstruction techniques, such as deep inferior epigastric perforator (DIEP), transverse rectus abdominis myocutaneous (TRAM), or latissimus dorsi (LD) flaps, were not included in this analysis. Procedures were executed between November 2024 and May 2025 at The Oncology Institute “Prof. Dr. Ion Chiricuta” Cluj-Napoca. Exclusion criteria included smokers and individuals with insufficient donor fat tissue.

The study was conducted in accordance with the guidelines of the Declaration of Helsinki and was approved by the Institutional Review Board and Ethics Committee of the Institute of Oncology “Prof. Dr. I. Chiricuță”, Cluj-Napoca, Romania (Assessment Report No. 356/17 November 2024).

### 2.1. Surgical Procedure

All patients in the study had previously undergone mastectomy followed by unilateral or bilateral breast reconstruction using prepectoral implants. Lipofilling was performed approximately 3 months following the initial reconstruction. Patients underwent between one and three lipofilling sessions.

The procedure followed Coleman’s structural fat grafting protocol. Donor sites were first infiltrated with Klein’s solution, composed of 1000 mL of 0.9% NaCl and 1 mL of epinephrine (1.0 mg/mL). After allowing 7 to 20 min for the solution to take effect, fat was manually harvested using a 50 cc syringe connected to a cannula. The collected fat was then purified using a sterile filtration system to remove blood residues and excess fluids, which involved a specialized connector. Once purified, the fat was injected into the target area using small-volume syringes attached to fine cannulas. Fat harvesting and injection were performed exclusively using Tulip Medical cannulas (Tulip Medical Products, Inc., San Diego, CA, USA). Adipose tissue was harvested with a Tonnard Harvester (Tulip Medical Products, Inc., San Diego, CA, USA) 3.0 mm (11G) × 20 cm (TONLL1120CF) following infiltration of tumescent solution using a Tumescent Infiltrator (Tulip Medical Products, Inc., San Diego, CA, USA) 2.1 mm (14G) × 20 cm (INFLL1420CF). Fat injection was carried out using a Tulip Injector (Tulip Medical Products, Inc., San Diego, CA, USA) 1.2 mm (18G) × 7 cm (INJLL1807CF).

Fat grafting was performed using 5 mL Luer-Lok syringes (Becton, Dickinson and Company, Franklin Lakes, NJ, USA), applying a multilayered microinjection technique. The injected fat volume ranged from approximately 80 to 150 mL per breast, depending on individual reconstructive requirements.

During the lipofilling procedure, the harvested fat was injected into distinct tissue planes, including the deep subcutaneous layer of the mastectomy skin flap and the prepectoral periprosthetic space surrounding the implant, without accessing the submuscular plane.

No acellular dermal matrix (ADM), mesh, or synthetic matrix was used in any case during prepectoral reconstruction.

No early complications attributable to fat grafting were observed; mild short-term local inflammatory reactions and transient ecchymoses were considered normal postoperative changes.

In the majority of cases, the placement of the implant was performed as a single-stage immediate reconstruction following mastectomy. However, in a few selected patients (depending on oncologic treatment sequencing, quality of skin envelope, or individual clinical factors) the reconstruction was delayed.

**Figure 1:**
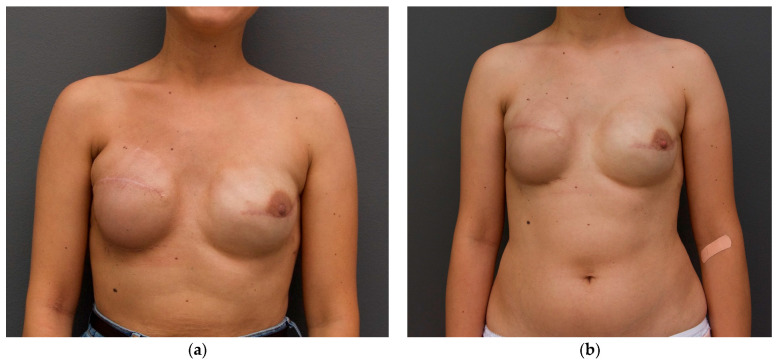
Illustrative example from a clinical case demonstrating the effect of autologous fat grafting on implant contour and rippling: (**a**) before lipofilling, pronounced rippling is observed in the upper pole of the reconstructed breast, with evident visibility of the implant margin, suggesting inadequate soft-tissue coverage; and (**b**) following autologous fat grafting, the breast contour is smoother, with a noticeable attenuation of the implant outline and a substantial reduction in rippling, reflecting improved soft-tissue coverage and contour refinement.

As an illustrative example from a representative clinical case, Figure 1 presents clinical photographs acquired before and after autologous fat grafting.

### 2.2. Evaluation of the Results

To accurately assess the results of hybrid breast reconstruction and the impact of fat grafting, patients were required to complete a modified questionnaire before undergoing lipofilling. This questionnaire, known as BREAST-Q, was developed by Memorial Sloan Kettering Cancer Center and The University of British Columbia (BREAST-Q Reconstruction Module, Version 2.0). The Romanian (RO) translation, completed in 2022 by a local academic team, was used under license from the BREAST-Q developers. It evaluates various aspects of breast reconstruction from the patients’ perspectives, including physical, psychological, and sexual well-being, as well as satisfaction with their breasts, the reconstruction outcome, and the care received. Data was collected both preoperatively and postoperatively, allowing for statistical comparison. Patients were asked to respond to the questionnaire again three months postoperatively, after receiving fat grafting. Patients were informed preoperatively that additional lipofilling sessions might be necessary to further optimize the aesthetic result. This approach facilitates a straightforward comparison of the two data sets and helps determine the effectiveness of fat grafting in enhancing the aesthetic, physical, psychological, and sexual outcomes of breast reconstruction with implants [13].

The structure and domains of the BREAST-Q questionnaire are complex, including 3 main categories of questions: group 1—aesthetic outcomes (questions 1, 2, 4, 5, 8, 9, and 12); group 2—medical/functional advantages (questions 3, 5, 10, 11, and 12); and group 3—medical procedure and overall care (questions 13, 15, and 16). The main topics of the questions are the following (Table 1):

- Satisfaction with Breasts, which grades the patients comfort with the appearance, symmetry, and general results of the breasts after being operated.
- Quality of Life, including subjects such as psychosocial, physical and sexual well-being, assessing the impact breast surgery has on patients’ lives.
- Satisfaction with Care—satisfaction with the medical care which was received, including communication skills with not only the surgeon, but also the medical team, and postoperative care.
- Physical Well-Being, which refers to symptoms and physical discomfort that patients might experience postoperatively.

**Table 1:** Classification and description of BREAST-Q items used in this study.

Question	Content	Category
Q1	Satisfaction with breast appearance	Aesthetic outcomes
Q2	Psychosocial well-being	Aesthetic outcomes
Q4	Sexual well-being	Aesthetic outcomes
Q5	Satisfaction with surgical outcome	Aesthetic outcomes Functional/medical outcome
Q9	Body image and self-confidence	Aesthetic outcomes
Q3	Physical symptoms and discomfort	Functional/medical outcomes
Q6	Pain or discomfort in the chest	Functional/medical outcomes
Q7	Difficulty with arm movement or daily activities	Functional/medical outcomes
Q8	Visibility and palpability of implant rippling	Functional/medical outcomes Aesthetic outcomes
Q10	Perception of breast sensation	Functional/medical outcomes
Q11	Psychological meaning of the surgery	Functional/medical outcomes
Q12	Preoperative fear about cosmetic result	Functional/medical outcomes Aesthetic outcomes
Q13	Information received from the surgeon	Satisfaction with care
Q14	Communication and support from the medical team	Satisfaction with care
Q15	Availability and responsiveness of the team	Satisfaction with care
Q16	Overall satisfaction with medical care	Satisfaction with care

The BREAST-Q is considered to be a gold standard for evaluating the outcomes in breast surgery, being used both in research and current clinical practice to further understand the perspectives of the patients, as well as to qualitatively improve the care provided.

### 2.3. Statistical Analysis

Statistical analysis was conducted to evaluate the changes regarding the BREAST-Q scores before and after lipofilling. Continuous data were expressed as means with standard deviations (SD). To assess the significance of pre-versus postoperative changes for each item, paired-sample *t*-tests were performed for normally distributed variables. For non-normally distributed variables or ordinal data, the Wilcoxon signed-rank test was used.

Normality of the data distribution was assessed using the Shapiro–Wilk test and inspection of Q-Q plots. A *p*-value < 0.05 was considered statistically significant, with more stringent significance considered at *p* < 0.01. All statistical analyses were performed using SPSS v26.0.

## 3. Results

Data were collected from a total of 96 patients who completed both the preoperative and postoperative versions of the BREAST-Q questionnaire. Of the 96 patients included in the study, 36 underwent bilateral procedures, primarily indicated due to positive genetic testing, while the remaining patients had unilateral reconstruction. To facilitate a structured analysis, the questionnaire items were grouped into three major categories: aesthetic outcomes, functional/medical outcomes, and satisfaction with care.

The summarized results for each individual question, including pre- and postoperative means, score differences, and statistical significance, are presented in Table 2. For clarity and interpretative value, the outcomes will be further detailed within each of the three defined categories.

### 3.1. Aesthetic and Psychosocial Outcomes

**Figure 2:**
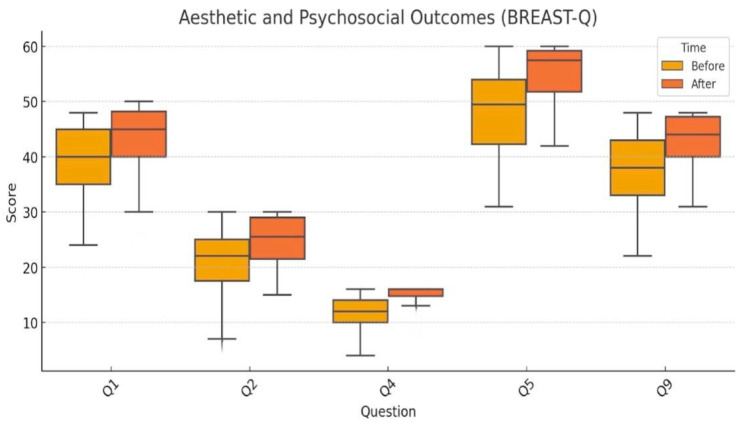
Aesthetic and psychosocial outcomes assessed by BREAST-Q. Boxplot comparison of preoperative and postoperative scores.

Significant improvements were observed in all parameters reflecting patients’ aesthetic satisfaction and psychosocial well-being (Figure 2):

- Q1 (Satisfaction with breast appearance): Analyzed using the paired *t*-test (*p* < 0.001), with a mean increase from 39.5 to 43.9.
- Q2 (Psychosocial well-being): Non-normally distributed, analyzed using the Wilcoxon test (*p* < 0.001), with improvement from 20.6 to 25.0.
- Q4 (Sexual well-being): Evaluated using the Wilcoxon test (*p* < 0.001); scores rose from 12.0 to 14.8.
- Q5 (Overall satisfaction with reconstructive outcome): *t*-test applied (*p* < 0.001); scores improved significantly from 47.5 to 54.8.
- Q9 (Body image and self-confidence): Wilcoxon test (*p* < 0.001), with a mean increase from 38.2 to 42.8.

### 3.2. Functional and Physical Outcomes

**Figure 3:**
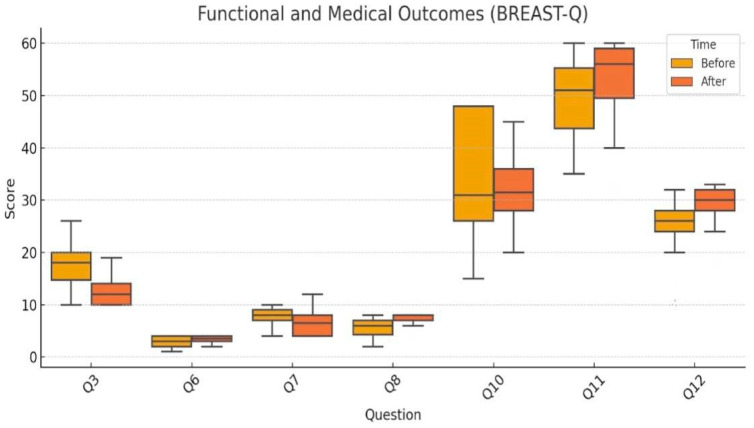
Functional and medical outcomes assessed by BREAST-Q. Boxplot comparison of preoperative and postoperative scores.

Each question related to functional and physical outcomes is represented in the following (Figure 3):

- Q3 (Physical symptoms/discomfort): Significant reduction from 17.6 to 12.6, analyzed using the Wilcoxon test (*p* < 0.001).
- Q6 (Pain): Modest improvement assessed via Wilcoxon test (*p* < 0.001).
- Q7 (Physical limitations): Also evaluated with Wilcoxon test (*p* = 0.01), showing a small but statistically significant improvement.
- Q10 (Breast sensation): Analyzed using the paired *t*-test, showing a nonsignificant decrease (*p* = 0.15), indicating preserved sensory function.
- Q8 (Rippling visibility): Evaluated only postoperatively, not subject to paired testing; mean score was 7.4, indicating acceptable aesthetic outcomes.

### 3.3. Satisfaction with Medical Care

High baseline satisfaction levels were maintained, or improved slightly postoperatively: Q13 (information received from the surgeon) and Q14–16 (communication, availability and professionalism of the medical team) showed consistently high scores pre- and postoperatively, with slight but statistically significant increases (all *p* < 0.05, tested with *t*-tests or the Wilcoxon test depending on normality).

## 4. Discussion

Lipofilling seems to have many advantages, making it adequate for common use in clinical practice. It is vital to take into account every patient’s preference and to provide adequate therapy for every case.

### 4.1. Interpretation of the Main Results

The BREAST-Q analysis revealed consistent and statistically significant improvements in most domains following lipofilling, suggesting a clear benefit of hybrid breast reconstruction over implant-only techniques.

The greatest absolute improvement was observed in Q5 (satisfaction with surgical outcome), where the mean score increased by 7.28 points (from 47.47 to 54.75, *p* < 0.01). This confirms that patients perceived lipofilling as a meaningful enhancement to their initial reconstruction, likely due to improved breast contour and correction of implant-related deficiencies.

Q1 (satisfaction with breast appearance) and Q2 (psychosocial well-being) also showed notable gains of 4.69 and 4.47 points, respectively (both *p* < 0.01), reinforcing the impact of lipofilling on aesthetic refinement and emotional recovery. The improvement observed in Q4 (sexual well-being) was more modest (+2.84 points), yet statistically significant, reflecting greater comfort and self-confidence in intimate contexts.

Interestingly, Q3 (physical discomfort) was the only item where a decrease in score indicated a positive effect (−5.03 points; from 17.59 to 12.56, *p* < 0.01). This suggests a meaningful reduction in postoperative symptoms such as tightness, pain, or tension, likely due to the soft-tissue cushioning provided by fat grafting.

While Q8 (rippling visibility) was evaluated only postoperatively, its mean score of 7.37 supports the conclusion that most patients experienced acceptable contour outcomes. However, variability in responses suggests that in some cases, further fat grafting may still be indicated.

The evolution of Q12 (anxiety about aesthetic outcomes) is particularly relevant: a mean increase of +3.65 points indicates that many patients initially feared suboptimal cosmetic results, which were later disproved by their postoperative satisfaction. This supports the psychological benefit of lipofilling in reducing anticipatory anxiety and restoring confidence.

Regarding Q10 (breast sensation), a mean postoperative decrease of 2.85 points was observed, which, while not statistically significant (*p* = 0.15), may reflect the known sensory limitations of implant-based reconstruction. The relatively stable scores observed in Q11 (psychological interpretation of surgery) (+3.71 points) support patients’ long-term acceptance and positive reframing of the surgical process.

Finally, the care-related items (Q13–Q16) remained consistently high, with small but significant improvements, suggesting that the medical team’s communication, accessibility, and professionalism were not only maintained but perceived as more valuable in the postoperative phase.

### 4.2. Comparison with the Existing Literature

Its medical advantages are highlighted in a meta-analysis realized by Chen et al., which encourages its use for treating postmastectomy pain syndrome but, still, further studies may be necessary to analyze the exact timing and volume to be injected [14]. Furthermore, another meta-analysis realized by Chen et al. encourages its use to alleviate fibrosis caused by radiotherapy, offering a natural and simple (minimally invasive) solution to the problem [15].

In addition, its safety, being a less invasive procedure, is currently recognized in the literature [1]. Data extracted from a meta-analysis conducted by Tukiama et al. (where 558 publications on the topic of oncological safety of lipofilling were analyzed) led to the conclusion that none of the outcomes had a statistically significant difference for disease recurrence after lipofilling sessions [16]. The same conclusion is reached by two other systematic reviews and meta-analyses by Al Qurashi et al. and Li et al., respectively, strongly pointing to the fact that fat grafting after breast reconstruction can be realized safely, which is contrary to the misconception of oncological risk of recurrence after lipofilling [17,18].

Medical implications are based on mesenchymal stem cells that are found in the adipose tissue that is harvested during lipofilling. These cells have the potential to differentiate into various cell types and play a crucial role in tissue repair and regeneration through their chemotactic, paracrine and immunomodulatory functions. Mojallal et al. demonstrated that adipose tissue improves skin quality by thickening the dermis and production of collagen fiber type 1 [19]. Rigotti et al. found that transplanting lipoaspirates enriched with adult adipose-derived stem cells (ASCs) is an effective treatment for degenerative, chronic lesions caused by oncologic radiation. Ultrastructural analysis of the radio-damaged tissue showed a significant reduction in the capillary bed. However, due to the angiogenic factors released by ASCs, lipofilling disrupted the cycle of vascular damage, ischemia, hyperpermeability, and fibrosis, promoting the growth of a microvascular network with a balanced ratio of adipocytes to capillaries [20].

A study by Ahmed Shaaban evaluated the effects of adding platelet-rich plasma (PRP) to processed fat for lipofilling in patients who underwent conservative breast surgery and experienced deformities or defects [21]. The study found that PRP-enriched lipofilling not only improves aesthetic outcomes, leading to higher satisfaction among both surgeons and patients, but also decreases local complications such as calcification, oil cyst formation, and fat necrosis at the recipient site [22]. These findings are consistent with other studies that have demonstrated PRP’s ability to stimulate nearby endothelial cells, enhance new capillary formation due to its high concentration of angiogenic growth factors, and prevent adipocyte apoptosis, thereby overcoming the challenge of insufficient neovascularization for fat survival [22].

In the past, there were widespread controversies regarding the use of lipofilling due to some complications such as fat necrosis, the formation of adipose cysts and calcifications that might interfere with the detection of breast cancer in early stages. The risk of malignancy that is associated with fat grafting still remains unclear because of the lack of randomized controlled trials and standardized techniques [7]. On the whole, the complication rate reported was 12.7%, liponecrosis being the most common of them. The incidence of fat necrosis was significantly variable across different studies, being influenced by the specific lipofilling technique which was used [14]. Typically, liponecrosis is asymptomatic, although some patients might experience ecchymosis or a palpable mass, locally. Mammographically, liponecrosis is often described as a radiolucent, rounded surface with a thin radiopaque border [17,18]. The existence of calcifications on mammography, especially in the early stages when they occur in a small area, may raise concerns related to potential breast cancer recurrence. On the other hand, a task force from the American Society of Plastic Surgeons (ASPS) from 2009 evaluated the safety and efficiency of lipofilling in a group of 283 patients. Follow-up, ranging from 1 month to 10 years, showed that most patients experienced satisfactory results. Based on these, the ASPS recommendations for safe and efficient application of fat grafting to the breast were elaborated.

A recent study conducted by Gigli S. et al. presents a 57-years-old woman who underwent lumpectomy for ductal breast carcinoma followed by lipofilling. The patient complains of a painful swelling in the outer quadrants of the operated breast. The ultrasonography of the breast revealed the presence of diffuse structural changes and many hypoechoic surfaces with acoustic shadowing, primarily located in the subcutaneous adipose tissue. Due to the unclear interpretation, another ultrasound was performed 3 months later and showed no improvement, pointing to further investigation with contrast-enhanced MRI, which was performed and confirmed diffuse structural modification with severe breast tissue edema, accompanied by several enhancing areas measuring up to 1 cm in diameter, with blurred margins. Despite a short course of the anti-inflammatory treatment, the lesions persisted, leading to a strongly suspected malignancy. An ultrasound-guided core-needle biopsy was performed, which revealed inflammatory infiltrate and fat necrosis, but no cellular atypia [23].

A systematic meta-analysis of the literature realized by Osswald et al. analyzed both prospective and retrospective studies that concentrate on imaging outcomes in patients with a history of breast cancer who received, in the past, one or more fat grafting procedures following oncologic breast surgery [24]. It is mentioned that each surgery or even traumatic procedure which is applied to the breast might cause inflammatory or remodeling processes. The authors state that fat necrosis occurs in up to 2.75% of all breast lesions. The most common cause is believed to be trauma which is followed by breast surgery accompanied by adjuvant radiotherapy. This article reported an average rate of 6.5% in biopsy, out of all imaging techniques. The local event rate measures 0.7% (after surgical oncologic breast therapies) when accompanied by lipofilling procedures. There seems to be an increased likelihood of biopsies in groups of patients who are monitored exclusively using ultrasound (6.9%) compared to when several imaging techniques are used (7.2–24%), in contrast to mammography alone (1%) [24]. It is also noted that the radiographic changes observed in fat-grafted breasts are, in general, sufficiently distinguishable from malignant alterations [25,26].

The process of preparing the fat tissue influences the risk of complication. A study conducted by Conde-Green A. et al. compared the influence of decantation, washing and centrifugation on adipocyte and mesenchymal stem cell content of aspirated adipose tissue. This study revealed that decantation turned out to be the least damaging of the fat-processing methods, because it preserves not only the integrity, but also the number of adipose cells. However, it also leads to a high concentration of contaminating blood cells, and respectively fewer mesenchymal stem cells (MSCs). Taking into consideration the critical role which regenerative cells have in graft survival, although centrifugation is more harmful to adipocytes, it effectively removed most blood remnants and provided the highest concentration of MSCs in the pellet. It could be isolated and, secondarily, added to other types of tissues to enhance survival. Nevertheless, the washing process removed most blood remnants and other toxic substances from the lipoaspirates and, additionally, proved to also be gentler to adipocytes to preserve an important quantity of adipose-derived MSCs [27].

Ultimately, Muntean et al. conducted a systematic review that analyzed 16 studies on hybrid breast reconstruction. A total of 730 patients were included, with a mean follow-up of 20.23 months. The overall complication rate was low (9%), with fat necrosis (2.7%) and capsular contracture (4.5%) being the most common. Immediate fat grafting during implant placement had the lowest complication rate (8%). Aesthetic satisfaction was high across studies, with an average score of 4.4 out of 5. Fat grafting improved skin quality, contour, and allowed for smaller implants, particularly benefiting irradiated patients. No oncologic recurrences were reported. These findings suggest that HBR is a safe and effective technique, offering improved cosmetic outcomes with low complication rates [28].

### 4.3. Limitations of the Current Study

The results presented in this study apply specifically to the hybrid implant-based approach and should not be extrapolated to other forms of autologous breast reconstruction.

The inclusion of patients from a single medical center further limits the sample diversity. Moreover, all participants voluntarily chose to undergo prophylactic mastectomy with immediate reconstruction, which may introduce selection bias. These individuals might exhibit distinct psychological traits, risk perception, or health awareness compared to those who declined surgery, potentially influencing the outcomes.

Postoperative data were gathered at three months after surgery, a duration considered adequate for the stabilization of early perceptions regarding aesthetic and psychological results. However, this period might not fully capture patient satisfaction or long-term quality of life. Shifts in body image, emotional adjustment, or late complications can arise beyond three months, necessitating extended follow-up to assess the sustainability of perceived benefits or dissatisfaction.

The responses collected via the BREAST-Q questionnaire are inherently subjective and may fluctuate depending on the patient’s emotional state at the time of completion. Satisfaction and quality of life are fluid concepts that evolve over time, complicating objective measurement. Therefore, individual variability might affect the consistency and reproducibility of the findings, despite the standardized format of the questionnaire.

Another limitation of the current study is that patient-reported outcomes were evaluated after only a single fat-grafting session. While several lipofilling procedures are often required due to partial fat resorption, the potential cumulative effect of additional sessions on patient satisfaction was not assessed. Thus, the improvements reported might underestimate the final aesthetic and psychosocial benefits achievable with staged lipofilling.

Moreover, adjunctive technologies (like water-jet–assisted liposuction) have been proposed to improve fat graft viability and aesthetic outcomes. In the present study, conventional liposuction and standard fat purification techniques were used consistently across all patients to ensure procedural homogeneity. Future studies comparing advanced harvesting devices with traditional techniques are further required to clarify their impact on graft survival and patient-reported outcomes.

## 5. Conclusions

Lipofilling performed as an adjunct to implant-based breast reconstruction was associated with significant short-term improvements regarding patient-reported aesthetic satisfaction and functional outcomes, as it was assessed using the BREAST-Q during the first postoperative evaluation. In this single-cohort, pre–post analysis, fat grafting contributed to elevated patient-perceived outcomes following reconstruction, without increasing early postoperative morbidity. Even though the observed improvements are in accordance with the concept of hybrid breast reconstruction, direct comparisons with implant-only reconstruction cannot be performed because of the absence of a control group. Thus, the findings should be interpreted as being based on patient changes rather than comparative superiority.

Hybrid breast reconstruction (involving lipofilling) makes it possible to offer both medical and aesthetic benefits for patients who require breast reconstruction. No recurrences have been registered in terms of oncologic safety, contrary to the popular belief of unsafety from this point of view. More research may be needed to draw conclusions on these findings, especially in areas with limited data. Nevertheless, due to its proven safety and simplicity, lipofilling is suitable for use in the current clinical practice whenever it is required, taking into consideration the opinions of both the patient and the plastic surgeon.

## Figures and Tables

**Table 2:** Summary of pre- and postoperative BREAST-Q scores and statistical analyses. The table presents the mean scores before and after lipofilling for each BREAST-Q item, along with the calculated difference and corresponding *p*-value. Statistically significant improvements (*p* < 0.01) were observed in most domains, particularly those related to aesthetic outcomes, physical symptoms, and satisfaction with care.

Question	Mean Before	Mean After	Difference	*p*-Value
Q1	39.0	43.69	4.69	<0.01
Q2	20.56	25.03	4.47	<0.01
Q3	17.59	12.56	−5.03	<0.01
Q4	12.0	14.84	2.84	<0.01
Q5	47.47	54.75	7.28	<0.01
Q6	2.84	3.38	0.54	<0.01
Q7	7.47	6.62	−0.85	0.01
Q8	-	7.37	-	-
Q9	38.19	42.84	4.65	<0.01
Q10	34.97	32.12	−2.85	0.15
Q11	49.41	53.12	3.71	<0.01
Q12	25.47	29.12	3.65	<0.01
Q13	11.35	9.18	−2.17	<0.01
Q14	54.34	57.62	3.28	<0.01
Q15	46.9	47.74	0.84	0.03
Q16	27.84	27.97	0.13	0.16

## Data Availability

Data are available upon reasonable request from the corresponding author.
